# Declining Public Health Protections within Autocratic Regimes: Impact on Global Public Health Security, Infectious Disease Outbreaks, Epidemics, and Pandemics

**DOI:** 10.1017/S1049023X20000424

**Published:** 2020-04-02

**Authors:** Frederick M. Burkle

**Affiliations:** Harvard Humanitarian Initiative, Harvard University & T.H. Chan School of Public Health, Cambridge, Massachusetts USA; Woodrow Wilson International Center for Scholars, Washington, DC USA; Institute of Medicine, National Academy of Sciences, Washington, DC USA; Prehospital and Disaster Medicine, WADEM, Madison, Wisconsin USA

**Keywords:** autocratic regimes, crisis management, epidemics/pandemics, global public health, public health emergencies

## Abstract

Public health emergencies of international concern, in the form of infectious disease outbreaks, epidemics, and pandemics, represent an increasing risk to the worldʼs population. Management requires coordinated responses, across many disciplines and nations, and the capacity to muster proper national and global public health education, infrastructure, and prevention measures. Unfortunately, increasing numbers of nations are ruled by autocratic regimes which have characteristically failed to adopt investments in public health infrastructure, education, and prevention measures to keep pace with population growth and density. Autocratic leaders have a direct impact on health security, a direct negative impact on health, and create adverse political and economic conditions that only complicate the crisis further. This is most evident in autocratic regimes where health protections have been seriously and purposely curtailed. All autocratic regimes define public health along economic and political imperatives that are similar across borders and cultures. Autocratic regimes are seriously handicapped by sociopathic narcissistic leaders who are incapable of understanding the health consequences of infectious diseases or the impact on their population. A cross section of autocratic nations currently experiencing the impact of COVID-19 (coronavirus disease 2019) are reviewed to demonstrate the manner where self-serving regimes fail to manage health crises and place the rest of the world at increasing risk. It is time to re-address the pre-SARS (severe acute respiratory syndrome) global agendas calling for stronger strategic capacity, legal authority, support, and institutional status under World Health Organization (WHO) leadership granted by an International Health Regulations Treaty. Treaties remain the most successful means the world has in preventing, preparing for, and controlling epidemics in an increasingly globalized world.

*“Honesty is worth a lot more than hope…”* The Economist, February 17, 2020.

## Part I: The Global Problem

Infectious diseases, in particular lower respiratory infections, remain the leading cause of death world-wide killing one-third of all people across all economic groups.^1^ While we acknowledge that the spread of infectious diseases is commonly exacerbated by human behavior, population density, inadequate public health protections, land and water use patterns and violations, increasing trade and travel, viral and bacterial mutations, as well as inappropriate use and increasing resistance to antibiotics, we rarely consider the purposeful lack of governmental leadership as a major factor in both the life and acceleration of preventable outbreaks, epidemics, and pandemics. Societies have experienced the benefits of advancements in public health infrastructure, prevention, and preparedness, yet these protections remain far from being globally understood, available, practiced uniformly, or free of political control.

What is increasingly common since the last one-third of the 20^th^ century is the thread of public health emergencies permeating, and often dominating, the consequences brought on by wars, conflicts, and large-scale disasters.^2^ Few are aware that wartime public health crises cause more deaths than weapons.^3,4^ Consistently in war, the public health protective threshold is destroyed and not recovered or maintained.^5^ Recovery is purposely ignored, resulting in increasing post-crisis mortality and morbidity indices that are characteristically ignored or denied, especially if they negatively impact political, ethnic, or religious groups whose views are contrary to the newly installed autocratic regime.

Ruger reminds us that authoritarian regimes suppress political competition and tend to have an interest in preventing human development, because improved health, education, and economic security mobilizes citizens to advocate for greater participation and more resources.^6^ Public health protections are literally invisible to populations; they are often taken for granted and applauded as great successes serving as propaganda ploys in public speeches.^7^ Although there has been scant investment in public health infrastructure and protections in all parts of the world, those countries suffer the most under autocratic regimes, especially where they have failed to keep pace with population growth and density.^8^ Currently, both the urban and rural environment of the 21^st^ century are being defined by deficient dwellings, aged and inadequate infrastructure, and insufficient capacity to respond to crises, especially in ensuring access to safe water, food, sanitation, and energy. Public health surveillance, the “continuous, systematic collection, and analysis of health-related data serve as an early warning system for impending public health emergencies, but compliance differs remarkably from one country to another.”^6,8^ Indeed, the direct and indirect mortality and morbidity resulting from these tragedies are the responsibility of the government in power, but are often the first to be ignored. Ecological and environmental protections and preservations, such as the continuous surveillance mandated by the World Health Organization (WHO; Geneva, Switzerland) of wet markets in China that launched SARS (severe acute respiratory syndrome) in 2008, is an example of a critical monitor that was ended prematurely. Only the reporting of three diseases (yellow fever, plague, and cholera) are currently binding under the International Health Regulations, and then some countries are unwilling to notify WHO fearing economic and political consequences.^9^

### Stable and Unstable Political Systems

The processes of political development, primarily as they apply to stable and unstable political systems and change, have always been dynamic, especially in crisis situations such as outbreaks of infectious diseases in less-developed countries. Crisis situations test the stability of political systems in revealing ways, placing extraordinary demands on the political leadership and the existing public health structure and processes of the country. In the absence of early and effective preparedness, societies may experience social and economic disruption, threats to the continuity of essential services, reduced production, distribution difficulties, and shortages of essential commodities. The WHO emphasizes a “whole-of-society” approach that emphasizes significant roles not only for the health sector, but also by all other sectors, individuals, families, and communities, in mitigating the effects of a pandemic.^10^ Developing such capacities is at the heart of preparing the whole of society for a pandemic. I assert that it is the loss of the whole of societyʼs concept, thinking, and participation that is systematically destroyed in autocratic regimes that contributes to why these political systems fail. They fail when citizens have no defined ownership, channels of communication, or are allowed to participate in any aspect of the disaster cycle (prevention, preparedness, response, recovery, or rehabilitation). They fail when citizens are not allowed a voice in the implementation of acceptable policies when the political system ceases to be viewed as responsive by individuals and groups making demands on it, and by what is considered inappropriate political behavior.

Infectious disease outbreaks have the uncanny capacity to question the status quo, catalyze smoldering unrest, and most importantly, reveal population-based public health imperfections.^11^ The “whole of society” which depends on a form of collaborative governance, which complements public policy, disappears and is seen only as the dictate of one person. Indeed, the negative influence on society, what I refer to a “societal mental health,” is out of proportion to their representation in society.^12^

The 2019 Democracy Index, compiled by the United Kingdomʼs Economist Intelligence Unit (London, UK) and published annually in *The Economist,* ranks countries according to political and civic freedom using five criteria: whether elections are free and fair, whether governments have checks and balances, whether citizens are included in politics, the level of support for the government, and whether people have freedom of expression. Nations are divided into “full democracies, flawed democracies, hybrid regimes [which include those exhibiting regular electoral frauds], and authoritarian regimes” where “political pluralism has vanished or is extremely limited.”^13^ The 2019 edition is considered as having the “worst average global score since the Index was introduced in 2006, driven primarily by regressions in Latin America and sub-Saharan Africa.^14^

Globally, this is the first time in the modern era where we have the fewest democracies. By ranking on how functional their political systems are, less than five percent of the worldʼs population live in a “full democracy.”^15^ Fewer countries can claim free and fair elections, checks, balances, and participation in their governments. Fewer nations offer freedom of expression or political participation in established political cultures. Rapidly established and increasingly prosperous autocratic regimes, many first drawn in by populist claims that enticed the masses of working-class and poor, are now firmly established by an economy ruled by dictators and oligarchs with unfettered political influence. The United States is now categorized as a “flawed democracy,” experiencing both undeniable presidential claims for more authoritarian rule, a population that increasingly claims loss of traditional liberties, and low esteem in which US voters hold their government, elected representatives, and political parties.^15^

### Characteristics of Autocratic Regime Leadership

Autocratic leaders demonstrate personality and behavioral characteristics that are remarkably consistent across borders and cultures.^16^ In great part, this is due to a common fault line from their adolescent development which becomes arrested cognitively and emotionally. While they may, at first glance, seems smart, they are not bright or capable of attaining abstract reasoning.^17^ This type of reasoning is required to formulate theories and understand multiple meanings crucial for reasoning. It demands generalizations, ideas, the ability to identify the relationship between verbal and nonverbal ideas, and to understand the multiple meanings that underlie an event, statement, or object; an example often cited is: “The Liberty Bell is not just a piece of American history, but is a symbol of freedom.”^18^ Concrete thinkers misinterpret many concepts like this and are compelled to reinterpret them in their own concrete manner in political speeches and legislative decisions.

Abstract thinking refers to a cognitive concept involving higher-order, or complex thoughts. To be able to think in an abstract manner implies that one is able to draw conclusions or illustrate relationships among concepts in a manner beyond what is obvious.^19^ Often the terms “abstract thought” and “concept formation” are used interchangeably. In the past, the term “fluid intelligence” has been used to refer to the ability to reason. The generation of concepts, or abstract ideas, indicates an ability to progress beyond concrete thinking. The concrete interpretation of a concept involves a focus on the salient, obvious characteristics. Progressing beyond the tangible characteristics in order to conceptualize theoretical relationships between items or processes involves abstract thought. Deeper meanings such as “freedom,” “equality,” “charity,” “love,” and “democracy” express ideas, concepts, or qualities that cannot be seen or experienced.^20^ They are considered only in the concrete sense as it applies to autocratic thinkers. The US Constitution would not be understood in the manner it was originally written as it is an example of a document that requires abstract thought and is either not read, understood, or interpreted concretely by a leadership that is completely self-serving. Concepts such as freedom and equal rights interpreted concretely become self-serving. Studies demonstrate that “persons with different value preferences apply different neurobiological strategies when facing a decision” and can help explain the fixed values that decisions are made that are independent of an actual situation.^21^ This stubbornness of thought and action is reflected in shared personality disorders of autocratic despots. Brain areas beyond those activated in actual moral dilemma situations were found to be involved. They are psychologically fixed, as illustrated by Muammar Gaddafi when he was being beaten to death by his own people, claiming up to the last minute: “but the people love me!”

Some of the well-known behaviors include cover-ups, exaggeration, and fabrication; fraud, omission, half-truth, perjury, and lies that come in various types, conveyed to exaggerate oneʼs credentials or get the attention that reflects their universal narcissistic disposition and constant needs. In great part, these behaviors are witnessed between all despots of the world. Despite the bad press lies get, and that many press agencies tally the daily lie numbers, most are ignored by political supporters in every country, particularly the ones that have spoken to avoid conflict, and as a show of collective support. Operationalization of narcissism is “dispositional” which accompany a “grandiose sense of self-importance, exhibitionism, entitlement, interpersonal exploitativeness, and a total lack of empathy.”^22^

Autocratic leaders:

[R]etain all power, authority, and control, and reserve the right to make all decisions; distrust their subordinateʼs abilities, closely supervise and control people under them; rarely delegate or empower subordinates; adopt one-way communication, do not consult with subordinates or consider their opinions; create a system of rewards and punishments; use threats and punishments and evoke fear; rarely concern themselves with developmental activities; and take credit for all the accomplishments.^23^

In truth, once in positions of power, only the most emotionally healthy and resilient can avoid the slide into psychopathology. For those with some of the personality attributes of sociopathy or psychopathy, the descent into deeper pathology may be beyond their ability to resist. Even their followers can become pathologically dependent.

Democracies characterized by individual freedom and liberty are rare. Throughout history, autocratic governments and tyranny have been the rule. Their lack of conscience and an inability to feel remorse are the underlying factors that are often viewed initially as charming, but soon reveal uncanny skills as master manipulators, skillful at lying and cheating. They have no capacity to feel guilt. Despite an incidence rate of three percent to five percent within the general population, and 25% of prison populations, it sometimes seems that they already rule the most despotic and populated areas of the world.^24^

## Part II: Pandemic Status of Countries under Autocratic Rule

As of February 20, 2020, 26 nations have WHO-confirmed cases of COVID-19 (coronavirus disease 2019) outside China. The Global Surveillance COVID-19 database centralizes all COVID-19 cases reported from outside China and is maintained at the WHO Headquarters in Geneva. Their data analysis is conducted daily to: “follow the transmission of the disease between countries; describe the characteristics of human-to-human transmission within clusters of cases; describe the characteristics of affected persons and their exposure history; and support the evaluation of public health measures implemented in response to the epidemic.”^25^

This study focuses only on countries under autocratic rule and describes the current status of public health preparedness and current responses. This review includes all countries run by one person or party with absolute power. Autocracy is a system of governance headed by a single ruler called an autocrat. Decisions made by the autocrat are not subject to legal restraints and the autocrat exercised unlimited and undisputed power.^26^ As of 2018, 50 nations are ruled by a dictator or authoritarian regime. Admittedly, democracy remains unsure in many countries, especially Africa, where dictators rising to power are increasingly likely. The study adds that: “Europe is home to one dictatorship, while three of them can be found in Latin America and South America. There are eight dictatorships in Asia, seven in the Eurasian region of the world, and twelve span territory from the northern parts of Africa to the Middle East.”^26^

### China

I cut my humanitarian teeth in China in the 1970s and 1980s when an unprecedented 83% of the population was suffering from poverty and malnutrition, one of the highest in the world. I was one of the few foreign physicians continually invited back under Maoʼs repressive regime. This allowed me an unprecedented view of Chinaʼs attempt to re-define what is the anthesis to the established global WHO requirements that guaranteed population-based public health protections. I taught basic public health management and reforms and helped establish emergency services to many hospitals. I was engaged in these activities while the government emphasized unprecedented industrial and economic development that contributed to rapid and “remarkable achievements” in the overall social and economic health of the population.

The incidence of poverty in China in 1981 declined from 85% to 27% in 2004, a reduction of slightly more than 600 million people, primarily accomplished through targeting rapid industrialization and village-based poverty.^27^ It also caused “twists and turns on the development of Chinaʼs public health” requirements, which lagged vastly behind industrialization. Public health was never given the same priority and failed to catch up with changes that required timely updating and adjustment of services.^28^

While it took time to recognize that China was on a path to also politically and economically redefine public health protections, infrastructure, and development, warnings directed at Chinaʼs new regional Centers for Disease Control (CDC; Beijing, China) fell on deaf ears. That same lack of coordination and collaboration remains evident today, placing China under a different microscope, one of greater scrutiny and judgment from the global community who sees their many poor health outcomes. Many of these poor outcomes are especially related to air pollution in re-defining hazardous air by WHO Standards as “acceptable,” and prompting many in China and the world to ask “at what price?”^29^

In 2010, there was water scarcity in two-thirds of Chinaʼs 600 cities, 80% had no sewage treatment facilities, the food security program was unsustainable, 90% of groundwater was polluted, and major rivers had their downstream microorganism ecology altered by chemicals and fertilizers dumped by industry and cities into the water. This resulted in new and re-emerging diseases.^30^ After identifying SARS origin from a wet market Civet source in August of 2016, President Xiʼs economic address, tied to security concerns, called for “full protection of peopleʼs health, stressing that public health should be given priority in the countryʼs development strategy.”^31^An independent survey of the Chinese citizenry two months later revealed that while the Chinese public agreed with Xiʼs need to promote Chinaʼs more influential role in the world, they raised grave concerns about environmental safety, numerous high-profile scandals regarding unsafe medical and food products, and water and air pollution.^32^ Chinaʼs story mirrors that of other developing countries in Asia, the fastest-growing region in the world, in that government spending on public health is inadequate and not focused on those who need it the most.

Studies in 2018-2019 confirm that 90 % of Chinaʼs groundwater is contaminated; tap water is not safe due to water contamination by the continued dumping of toxic human and industrial waste, because oxygen levels have obliterated normal organisms in all major rivers and only algae continue to flourish. Air quality remains “very unhealthy” and continues to have a major toll on public health, resulting in 350,000 to 400,000 premature deaths.^33,34^ It remains unclear whether China will ever meet its air pollution goals, letalone participate in global climate commitments to reduce carbon emissions.^34^

No one in global public health was surprised to learn that once again a wet market animal, not suited for human consumption, was probably responsible for this yearʼs COVID-19 pandemic. However, Chinese researchers now stress that the virus did not originate in the wet market, but was transferred from elsewhere, on December 8th and again on January 6th.^35^ Transmission could have begun in early December or late November, admitting the world-wide spread could have been limited had the earlier alerts been implemented.

After SARS in 2002, external pressure has also impacted on the development of Chinaʼs public health.^36^ During the SARS outbreak, the WHO directly told the Chinese government in its mission report in April 2003 that “[t]here was an urgent need to improve surveillance and infection control” in the country.^37^ Two years later, in a joint report issued by State Development Research Center (Beijing, China) and WHO, the Chinese government officially admitted its health care system was failing, and it needed to improve its disease surveillance system at the local wet market levels if they were to be seen as a “responsible state.”^38^

In December of 2019, the first cases of COVID-19 were diagnosed in Wuhan, the capital of Hubei Province, and rapidly expanded. For two weeks, the existence of a novel rapidly expanding virus was known to President Xi. Unconscionably, China arrested, jailed, and punished physicians and journalists who defied government attempts to silence the truth of the virus. Moreover, the government ceased to enforce the timely flow of crucial public health information, delaying both critical medical care, its obligations to the WHO, and the sacred paradigm of human interaction with a disease that collectively defines “freedom of speech.”^39^ Andrew Price-Smith put the same point succinctly post-SARS, stating that “while the SARS epidemic may have generated moderate institutional change at the domestic level, it resulted in only ephemeral change at the level of global governance.”^40^ In other words, national sovereignty is still of paramount importance for the Chinese leadership. Because of its sensitivity to foreign interference into its internal affairs, the Chinese government has not yet formally or officially endorsed the notion of “human security.”^40^ While China has embraced multilateral cooperation in a wide array of global health issues, its engagement remains “state-centric.”^37,38^

The SARS event not only exposed a fundamental shortcoming of Chinaʼs public health surveillance system, as well as its single-minded pursuit of economic growth since the late 1970s, but also forced China to realize that, in the era of globalization, public health is no longer a domestic, social issue that can be isolated from foreign-policy concern.^37^ Having no tolerance in ceding its supreme authority, the central government has adopted a multi-faceted attitude towards its civil society organizations. While Beijing shows its willingness to cooperate with a wide array of actors inside China, it refuses to let its domestic nongovernmental organizations (NGOs) and activists establish direct links with their counterparts overseas.^37,41^

China was openly accused of a cover-up with SARS, and few professionals are confident that anything has changed.^42^ Chan maintains that while “it is still uncertain whether this sovereign concern will trump the provision of global public good for health. Nevertheless, in a highly globalizing world, infectious diseases know no border. While China is seeking to adhere as much as possible to the underlying norms and rules of global institutions,” reemphasizing that China after SARS “perhaps [needs] to reframe health as a global public good that is available to each and every individual of the world, rather than merely as an issue of concern to nation-states.”^37^

In a rare openness, rarely seen before, the normally secretive Xi admitted at a meeting to coordinate the fight against the virus that China must learn from “obvious shortcomings exposed during its response.” Yet given the second-guessing that always surfaces in these tragedies, “it cannot be denied that the Chinese government tried to control the narrative, another sign of irrational hubris, and as a result, the contagion was allowed to spread, contributing to equally irrational fear.” A China researcher for Human Rights Watch (New York USA) noted: “authorities are as equally, if not more, concerned with silencing criticism as with containing the spread of the coronavirus.…repeating a pattern seen in past public health emergencies.”^43^ Although less clumsy than with SARS, the government kept all non-Party groups that could have helped prevent the spread of the virus out of the loop.^44,45^ Chinaʼs religious groups who “reflect the countryʼs decades-long revival and feeling among many Chinese that faith-based groups provide an alternative to the corruption that has plagued the government” are being ignored.^46^ Will this just be a temporary stay as it was post-SARS, or is China capable of adopting, without conditions, the WHO public health requirements they have ignored to date?

### North Korea

North Korea, the most sealed-off country in the world, has literally shut down all borders and communications on COVID-19, denying, according to their propaganda channels, the existence of any cases or deaths. This is unusual as it sits between China and South Korea, which have recorded the largest numbers of cases. Researchers state it is “unlikely that North Korea is free of COVID-19.” South Korean media reported that Kim Jong Un, the North Korean leader, had an official executed for violating the quarantine after the official returned from a trip to China. This may or may not be true since such reports have proved dubious in the past. North Korea press outlets claim that “not one novel coronavirus has emerged;” yet South Koreaʼs Unification Ministry (Seoul, South Korea), in charge of inter-Korean relations, reported to the WHO that North Korea had tested 141 suspected cases of coronavirus and all came up negative.^47^ Nevertheless, South Korean media, relying on anonymous sources, report cases of COVID-19 in North Korea, some of them fatal, according to John Linton, head of the International Health Care Center at Severance Hospital in Seoul: “Through private sources, they’re asking for disposable gowns, gloves, and hazmat suits, which are undoubtedly lacking,” he says. “So something is going on, otherwise they wouldn’t be asking for this.”^47^

North Korea relies on China for more than 90% of its trade. Researchers admit that while health indicators have improved in the two decades since the countryʼs 1990s famine, during which hundreds of thousands of people starved to death,^48^ but there are still major problems. In the 1990s, Amnesty International (London, UK) detailed a crumbling health care system in North Korea, a nation unable to feed its population, and, in violation of international law. North Korea refused to cooperate with the international community to receive food. Levels of malnutrition, maternal health, and tuberculosis (TB) are chronic problems, but a lack of accurate data on HIV/AIDS and hepatitis B present cause for alarm. Health indicators have improved in the two decades since the countryʼs 1990s famine, but major problems still exist. Whereas communicable diseases account for a large proportion of the disease burden, there are very few opportunities to better understand and control them.^49^ While health infrastructure has improved, capacity is low and the health system is chronically under-resourced. North Korea has allowed for United Nation (UN) interventions, primarily focused on sustainable development, but this has been on North Koreaʼs terms, a demand not unusual for autocratic regimes.^50^

In 2014, the report of the UN Commission of Inquiry on Human Rights in the Democratic Peopleʼs Republic of Korea (DPRK) concluded that: “20 years after humanitarian agencies began their work in the DPRK, humanitarian workers still face unacceptable constraints impeding their access to populations in dire need.”^51^ The report found that the DPRK has “imposed movement and contact restrictions on humanitarian actors that unduly impede their access.” The DPRK has “deliberately failed to provide aid organizations with access to reliable data, which, if provided, would have greatly enhanced the effectiveness of the humanitarian response and saved many lives.” The North Korean government “continually obstructed effective monitoring of humanitarian assistance, presumably to hide the diversion of some of the aid to the military, elite, or other favored groups, as well as to markets.” In summary, the report stated:

In this tightly controlled political climate, international humanitarian staff often have to make compromises. Some point out privately that it is unrealistic to try to uphold humanitarian standards in an environment as difficult as North Koreaʼs. They try hard to come up with ways to make their aid sustainable for the North Korean people, but their plans are not always accepted.^51^

Although the knowledge of public health has improved in recent years, 18 million people are dependent on a public distribution system of food rations and more than 10 million are under-nourished.^52,53^

### Iran

Early in the COIVID-19 crisis, Iran introduced containment measures that China had instituted placing tens of millions of people under lockdown. Yet, Iran has confirmed 43 infections and eight deaths, and appears to have entered the epidemic phase of the disease. Pakistan and Turkey announced the closure of land crossings with Iran, while Afghanistan said it was suspending travel to the country. Four new COVID-19 cases surfaced in Tehran, seven in the holy city of Qom, two in Gilan, and one each in Markazi and Tonekabon. As of this writing, several reports from the cities in the south, west, center, and north of Iran indicate cases testing positive for COVID-19.

The Iranian Minister of Health stated that the origin of the virus was in Qom, where infected Chinese nationals and Iranians who traveled to China during its pandemic were diagnosed. Reports suggest that a minimum number of cases is between 1,000 to 1,500, with additional unofficial reports of deaths from Hamedan, Saveh, Tonekabon, and Tehran, suggesting that the government under-reports the number of positive cases.^53^

The health ministry ordered the closure of schools, universities, and cultural centers across 14 provinces. All sport and cultural events were shut down for two weeks and all educational public exams were postponed. Unfortunately, many health workers and physicians are among newly infected cases, including the Deputy Health Minister.^53^ The country suffers a lack of basic equipment such as masks and disinfecting materials, even in health care centers. People are in a panic due to a lack of access to protective materials and angry over the government cover-up.^54,55^ Personal contacts in Iran, unfortunately, report that: “there is a major concern of misinformation because people do not trust the governmental information, opening the doors for rumors and more misinformation.”

Paul Hunter, professor of medicine at Britainʼs University of East Anglia (Norwich, England), said the situation in Iran has “major implications” for the Middle East. “It is unlikely that Iran will have the resources and facilities to adequately identify cases and adequately manage them if case numbers are large.”^56^

### Turkey

As of this writing, Turkey has not reported any COVID-19 infections. The government has closed its border with Iran, introduced health checks from Iran, and are turning back travelers. Yet travel from Turkey to Iran continues. Turkey is strategic in its geographic position. It is bordered by eight countries, is the intersection point of Asia, Europe, and Africa, making it one of the most strategic countries in the world. With its geopolitical position, Turkey is a unique bridge between eastern and western civilizations and between all religions.^57,58^ I bring up Turkey because that nation also has one of the most autocratic regimes in the world, which has mastered control over the population and media. The government has a pattern of undercutting criticʼs claims, accusing the opposition of having ulterior motives, and systematically undercutting the independence of the rule of law.^57^ Recep Tayyip Erdoganʼs one-man rule–control all executive, legislative, and judicial functions by imprisoning critical journalists and destroying what was left of the free media. He has arrested teachers, police, and government workers.

Erdogan must be in control of the narrative on all issues, including health.^59^ After the lessoned learned in China with one non-medical voice controlling all news on COVID-19, a similar false narrative, seen with all dictators, may again occur. Health differences with their northern European Union (EU) neighbors were a concern that delayed accession talks for full membership in the EU in 2005. One-half the population is made up of secular and liberal Turks who wish to restrain Erdogan and his abuse of power.^59^

### African Nations

Autocratic or authoritarian regimes–dictatorships–have been a dominant form of governance in Africa for many years. In the second decade of the 21st century, one concern is that they may hinder the attainment of one of the UNʼs crucial sustainable development goals.

In the last three years, analysts say that African countries have registered an overall decline in the quality of political participation and rule of law. The British Broadcasting Corporation (BBC; London, UK) recently reported that “more and more elections are being held in Africa.” However, analysts dismiss many as being “lawful but illegitimate.” Although studies show a majority of Africans still want to live in democracies, an increasing number are looking to alternative, autocratic models.^60^ African countries, in the last three years, have registered an overall decline in the quality of political participation and rule of law; analysts say: “Today there are almost the same number of defective democracies (15) as there are hardline autocracies (16), among the continentʼs 54 states,” Nic Cheeseman, Professor of Democracy at Birmingham University (Birmingham, England), concludes from his analysis of the last three years.^60^ Nigeria is among those listed as a “defective democracy,” which underscores the importance of recognizing fragile political parties in Africa. Recent elections in Nigeria illustrates this.^60^ Nigeria is seen as an emerging democracy often found in newly emerging states, and established democratic regimes existing in states with long traditions of uninterrupted sovereignty.^60^

Most critically, many autocratic African countries have been thrown into an inescapable political mix with China because of Chinaʼs close economic ties with multiple African countries. This economic dependence on China has grown so fast that it has critical future implications. The rapidity in which China has launched its massive continent-wide initiatives has been lost on many. The COVID-19 pandemic has awakened scholars to revisit its impact on Africa, where the worldʼs most powerful autocratic regimes exist.^61^ As of 2012, the African continent was home to more than 1.1 million Chinese immigrants.^62^

From 2001 to 2017, Chinaʼs Africa strategy began to solve over-population, pollution, and the poor economy in Africa and other developing countries. China offered sizeable loans to finance infrastructure projects, which incurred major debts for many third world nations, but especially Africa. These loans have changed the cultural and ethnic landscape of many struggling nations.^63^ The building of African ports, highways, and railways, all with Chinese money, have primarily corporate-level intentions, not the daily welfare of the populations. On the surface, these sound infrastructure projects are what Africa legitimately sees as necessary for progressing out of poverty. But on closer examination, they serve Chinaʼs ambitions to write the rules of the next stage of what they define as “globalization.”^64^ Of major concern is that these African countries are now defaulting on the loans, primarily funded by countries other than China, for daily external assistance and survival. The very predictable failures of the African countries to pay back the loans have entrapped African nations even further: “China, as the only major creditor in Africa, won’t be far away from taking hold of virtually every industry in Africa.”^65^

According to the agreements set up by China, the African nations can repay loans with natural resources such as oil. Yet, the defaulted loans made for constructing ports that were not productive are already owned by China. Chinaʼs massive “Belt & Raid Initiative” was designed to link up to 70 countries, all tied to Chinaʼs multiple infrastructure contracts and investments. Overland routes for roads and rail transportation guarantee that most countries involved will never be able to fully pay the loans and will remain dependent on China for their trade economies in the coming years. This receives very little attention in the Western press. In 2017, *Forbes* reported that China now owns international port holdings in Greece, Myanmar, Israel, Djibouti, Morocco, Spain, Italy, Belgium, Cote d’Ivoire, Egypt, and about a dozen other countries.^66^

In 2018, China took control of Kenyaʼs largest port after that nation defaulted on its unpaid Chinese loans. China wants everything from Africa–its strategic location, its rare earth metals, and its fish. This leaves African nations forever indebted to Beijing. Over one million Chinese now work in Africa, with one author citing that Africa is “Chinaʼs Second Continent,”^67^ but the actual long-term impact of these many transient workers on Africanʼs future is mixed. One author summarized that “on closer examination, Chinaʼs ambition is to write the rules of the next stage of globalization. This suggests that Beijing will not accept anything less than being the dominant landlord, one that is autocratic and mimicking the current authoritarian regime in China. China wants Africaʼs resources and its maritime roads for Beijingʼs large military presence.” This is evident from the fact that Chinese troops and weapons outnumber all other countries, especially the US, which is decreasing its military footprint. China formally launched its first overseas military base in Djibouti, where it constructed strategic ports, an electric railway, logistics, and intelligence facilities.^68^ But in all their projects, they focus on highways, ports, dams, and public networks, such as electric grids, not public health infrastructure. Military might is their priority, a model taken from the US over the past two decades. While the US today is trimming down its military presence in Africa, China is increasing theirs.

From the outset, China and heads of State from 53 African countries met to implement eight major initiatives to strengthen the cooperation between China and Africa. Some of the initiatives included industrial, trade, and cultural promotion, with public health ranking as a top priority for the China-Africa health cooperation plans. In 2017, there were 1,050 health professionals from China working in all 53 African countries, focusing on public health training and disease-control programs centered on emerging infectious diseases, malaria, HIV/AIDS, and health informatics, in collaboration with Africa CDC (Addis Ababa, Ethiopia), US CDC (Atlanta, Georgia USA), and other global partners.^69^ What remains a contradiction is the strong health priorities of the China-African Cooperation, which emphasizes many health initiatives that mainland China currently lacks. But China looks to the future and its survival. As they say in their next phase of “globalization,” African economic dominance will be necessary for Africaʼs survival.^69^ What political regime will rule at that time is questionable, but will probably be autocratic across China, Africa, and other countries that currently face a potential military takeover by China, such as Cambodia and Myanmar. In the meantime, WHO and other regional and country public health experts are concerned the “fragile” health systems in most African countries will not be able to cope if coronavirus takes hold on the continent. Even China, with its larger pool of technical and financial resources, appears to be struggling to contain the virus.^70,71^

### Russia

For all the advances in weaponry, including the first hypersonic missile, the poor-quality of public health directly “undermines the countryʼs economic development.” Their aging population and declining birth rates contribute to the low overall health status and low life expectancy. More than two million Russian men are considered to be HIV positive and extremely high multi-drug resistant TB persists. The direct connection between the public health crisis and Russiaʼs economic potential is clear. It is generally accepted that the highly productive educated soviets leave the country largely for reasons having to do with the deteriorating political freedoms in the country. Failure to tackle Russiaʼs huge public health problems is likely to exacerbate the brain drain already underway. It is estimated that up to 2010, more than 1.25 million Russians emigrated. That represents an even greater number than those who left after the collapse of the Soviet Union.^72,73^

Russia reported its first two cases of COVID-19 and said the infected people were Chinese citizens who have since recovered. The first three Russian citizens have also been infected with COVID-19 onboard a quarantined cruise ship in Japan. Around 2,500 people arriving from China have been ordered/placed under quarantine for COVID-19 and monitored by the Russian capitalʼs facial-recognition technology.^74^ Their quarantine measures have mimicked other nations and appear robust, but remain challenging to the economy and sustainability.

The one Achilles heel in Russiaʼs public health is the abominable rise of infectious diseases such as TB and AIDs. Public measures for their control in Russia are insufficient, mainly because of the lack of funding for treatment, vaccine prophylaxis, and health education. Tuberculosis has become an epidemic in a country where it was once a rarity. Immunity is down because of poverty, too little food, and difficult access to health care. Russian doctors are worried that the TB epidemic could lead to epidemics of another disease. Today, TB is endemic in Russia, and there is a rising incidence of multi-drug-resistant strains of TB.^75^ Like other autocratic regimes, Russiaʼs “political model” of globalization that feeds transnational research and treatment of infectious diseases is seriously flawed and must take responsibility for the prevention of the spread of infectious disease beyond their borders accelerated by enhanced migration.^76^ What this reveals are cautious doubts about whether Russia, combined with shortages of medical supplies and inadequate standards that further highlights a number of public health challenges for the country, has the public health and political capacity to manage a serious COVID-19 epidemic.

### Additional Dictators

The Borgen Project, which addresses poverty and hunger, focuses on the leaders of the most powerful nations addressing the need to deal with poverty as a consequence of their dictatorial rule. It is repeated here as it serves as an objective measure of the consequences of a despotic rule, as well as an indication of the physical and emotional state of populations that might not survive the additional insult of an infectious disease:^76^

**Table:** 

Country:	2018 Poverty rate:
North Korea	40.0%
Burundi	64.6%
Venezuela	82.0%
Syria	82.0%
Chad	46.7%
Rwanda	39.1%
Turkey	21.9%
Equatorial Guinea	76.8%

### United States

The United States, now designated a “flawed democracy,” is showing increasing authoritarian rule and threats to basic health protections, especially in combatting communicable diseases. Most concerning is the presidentʼs embrace of authoritarian leaders and the real possibility of major pandemic prevention funding, including the Emergency Reserve Fund, which is designed to be “quickly deployed to respond to pandemic outbreaks.” President Trump has mimicked other autocratic leaders’ positions in managing any serious outbreak. He has praised President Xiʼs rulings and failed to comment on the Chinese rulerʼs decision to punish physicians for grossly delaying international warnings and calling attention to the public health threat for which Xi was totally responsible. Trumpʼs narcissistic personality will force him to be defensive and again lie to save face. Peter Navarro, Trumpʼs senior trade advisor, is quoted: “This delay allowed the virus to proliferate much faster than it otherwise would have and reach other countries that it might otherwise have not.”^77^ Trump does not possess the knowledge base or intellectual capacity to be the spokesperson for any North American outbreak.

Most critically, Trump has set up a narrative that will impair the USʼs ability to manage any serious outbreak. He has argued for cutting spending for the CDC, National Institutes of Health (NIH; Bethesda, Maryland USA), and Medicare directly related to communicable diseases and will directly hinder any public health response. He is oblivious to the current status of emergency medicine departments in all hospitals, rural and urban, which are currently overloaded and have no beds for influenza patients. Patients must remain in emergency rooms until critical care beds open somewhere in the system, and that may take days. In no manner is our current health system capable of handling a serious outbreak, and the failure to begin a dialogue with practicing medical professionals is being ignored.

## Conclusions

Lipsitch predicts that some 40%-70% of the worldʼs population will be infected this year.^78^ Despite political claims, a vaccine is more likely seen within a year or two at best.^79^ It is no longer realistic to expect the management of these gaps in infectious disease outbreaks, especially those that threaten to be epidemics and pandemics, are to be capably managed in their present state of willful denial and offenses by many countries, especially those that are ruled by authoritarian regimes.^80^

Despite resistance to globalizationʼs health benefits that would markedly benefit the global community during these crises by authoritarian regimes, in 2015, I called for a new WHO leadership granted by the International Health Regulations Treaty that has consequences if violated. I stated:

The intent of a legally binding Treaty to improve the capacity of all countries to detect, assess, notify, and respond to public health threats are being ignored. While there is a current rush to admonish globalization in favor of populism, epidemic and pandemics deserve better than decisions being made by incapable autocrats. During Ebola, a rush by the Global Health Security Agenda partners to fill critical gaps in administrative and operational areas was crucial in the short term, but questions remain as to the real priorities of the global leadership as time elapses and critical gaps in public health protections and infrastructure take precedence over the economic and security needs of the developed world. The response from the Global Outbreak Alert and Response Network and foreign medical teams to Ebola proved indispensable to global health security, but both deserve stronger strategic capacity support and institutional status under the WHO leadership granted by the [International Health Regulations] Treaty. Treaties are the most successful means the world has in preventing, preparing for, and controlling epidemics in an increasingly globalized world. Other options are not sustainable. Given the gravity of on-going failed treaty management, the slow and incomplete process of reform, the magnitude and complexity of infectious disease outbreaks, and the rising severity of public health emergencies, a recommitment must be made to complete and restore the original mandates as a collaborative and coordinated global network responsibility, not one left to the actions of individual countries. The bottom line is that the global community can no longer tolerate an ineffectual and passive international response system. As such, this Treaty has the potential to become one of the most effective treaties for crisis response and risk reduction world-wide. Practitioners and health decision-makers world-wide must break their silence and advocate for a stronger Treaty and a return of WHO authority. Health practitioners and health decision-makers world-wide must break their silence and advocate for a stronger Treaty and a return of WHOʼs undisputed global authority.^81^

Will Chinaʼs unilateral decisions just be a temporary stay as it was post-SARS, or is China capable of adopting, without conditions, the WHO public health requirements they have so far ignored?

Autocratic leaders in history have a direct impact on health security. Dictatorships, with direct knowledge of the negative impact on health, create adverse political and economic conditions that only complicate the problem further. This is more evident in autocratic regimes where health protections have been seriously and purposely curtailed. This summary acknowledges that autocratic regimes are seriously handicapped by sociopathic narcissistic leaders who are incapable of understanding the health consequences of infectious diseases or their impact on their population. They will universally accelerate defenses indigenous to their personality traits when faced with contrary facts, double down against or deny accurate science to the contrary, delay timely precautions, and fail to meet health expectations required of nations under existing International Health Regulations, laws, and Epidemic Control surveillance.^82^

Kavanaughʼs *Lancet* editorial initially praised Chinese tactics that reflected a level of control only available to authoritarian regimes. As days and weeks passed, it revealed a government that inherently became victims of their own propaganda based on “need to avoid sharing bad news.” He concluded that authoritarian politics inhibited an effective response, and that openness and competitive politics favor a strategically fair public health strategy.^83^ Democratic nations in comparison to autocratic regimes recognize that public health fundamentally depends on public trust.^84^ The WHOʼs China Joint Mission on Coronavirus Disease report has applauded Chinaʼs eventual response capability and capacity with strict measures to interrupt or minimize transmission chains with extremely proactive surveillance, rapid diagnosis, isolation tracking, quarantine, and population acceptance of these measures, to implement the measures to contain COVID-19 within the country.^85^ It must not be forgotten that Chinaʼs authoritarian rule “put secrecy and order ahead of openly confronting the growing crisis and risking alarm or political embarrassment,”^86^ arrested and compelled Dr. Li Wenliang to sign a statement that his warning constituted “illegal behavior,” all of which delayed a concerted public health offensive that led to his death.^86^ This was an “issue of inaction” that would have contained COVID-19 within China and remains a potent symbol of Chinaʼs failures.^86^ There is no evidence that the authoritarian regime has or will change to prevent this from happening again.^87^ I suspect Chinaʼs sophisticated censorship and propaganda systems will outlast any public health improvements.
